# Bitter melon extract suppresses metastatic breast cancer cells (MCF-7 cells) growth possibly by hindering glucose uptake

**DOI:** 10.17912/micropub.biology.000961

**Published:** 2023-09-05

**Authors:** Abhinav Kakuturu, Heeyun Choi, Leah G Noe, Brianna N Scherer, Bikram Sharma, Bilon Khambu, Bhupal P Bhetwal

**Affiliations:** 1 Division of Biomedical Sciences, Marian University College of Osteopathic Medicine, Marian University - Indiana, Indianapolis, Indiana, United States; 2 Department of Biology, Ball State University, Muncie, Indiana, United States; 3 Department of Pathology and Laboratory Medicine, School of Medicine , Tulane University, New Orleans, Louisiana, United States

## Abstract

Breast cancer is one of the most commonly diagnosed cancers among women, however the complete cure for metastatic breast cancer is lacking due to poor prognosis. There has been an increasing trend of dietary modifications including consumption of natural food for the prevention of cancer. One of the popular natural foods is bitter melon. Bitter melon grows in tropical and subtropical areas. Some of the beneficial effects of bitter melon towards disease including cancer have been reported at the whole body/organismal level. However, specific cellular mechanisms by which bitter melon exerts beneficial effects in breast cancer are lacking. In this study, we used a human metastatic breast cancer cell line, MCF-7 cell, to study if bitter melon alters glucose clearance from the culture medium. We co-cultured MCF-7 cells with bitter melon extract in the presence and absence of supplemented insulin and subsequently measured MCF-7 cells viability. In this study, we report a noble finding that bitter melon extract exerts cytotoxic effects on MCF-7 cells possibly via inhibition of glucose uptake. Our findings show that insulin rescues MCF-7 cells from the effects of bitter melon extract.

**Figure 1. Insulin rescues human metastatic breast cancer cells (MCF-7 cells) from cytotoxic effects of bitter melon extract (BME):**
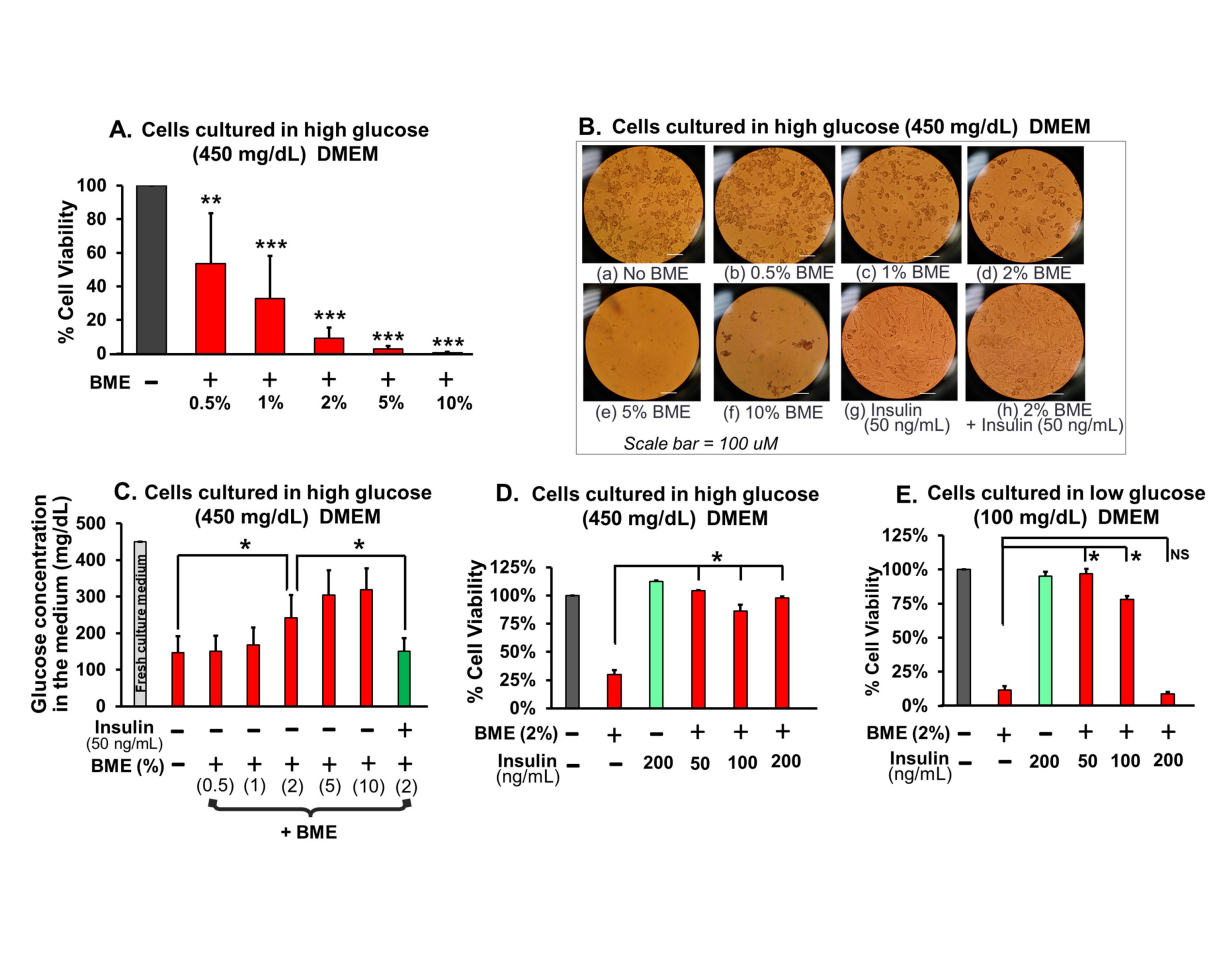
(A) MCF-7 cell viability determined by using Trypan blue assay. Different doses of bitter melon extract (BME) was used [0.5% to 10% (v/v)]. As mentioned in the description section, the bitter melon extract was obtained by grinding whole bitter melon (with seeds in the bitter melon). Cumulative data of average % viable cells (±SD) with N=3. Average % significantly different from the no BME control cells (**P < 0.01, ***P < 0.001), one-way ANOVA. (B) Microscopic images of MCF-7 cells (cultured in low glucose DMEM medium) after washing with 1X PBS. (a) control cells with no BME added to the cell culture. (b), (c), (d), (e), and (f) are the MCF-7 cells co-cultured with different doses of BME(v/v %). Note that (f) contains some cell debris from dead cells that were not completely washed during PBS wash. These are not live cells. (g) Cells co-cultured with insulin (50 ng/mL). (h) Cells co-cultured with 2% BME and insulin (50ng/mL). (C) Glucose concentration remaining in MCF-7 cell culture media. The cells were cultured in high glucose (450mg/dL) DMEM medium. Glucose remaining in the medium was measured by a glucometer after the 48 hours co-culturing with different doses of BME. The green bar represents cells co-cultured with BME (2%) and insulin (50ng/mL). Cumulative data of average amount of glucose in mg/dL (±SD) with N=5. Average mg/dL significantly different (*P < 0.01), one-way ANOVA. (D) Cell viability of MCF-7 cells cultured in high glucose (450mg/dL) DMEM medium . Cell viability was determined by using MTT assay. Cells were co-cultured with 2% BME, insulin (200 ng/mL), or both 2% BME & insulin (50 or 100 or 200 ng/mL). Cumulative data of average % cell viability (±SD) with N=3. Average % significantly different (*P < 0.01), one-way ANOVA. NS; Non significant. (E) Cell viability of MCF-7 cells cultured in low glucose (100mg/dL) DMEM medium. Cell viability was determined by using MTT assay. Cells were co-cultured with 2% BME, insulin (200 ng/mL), or both 2% BME & insulin (50 or 100 or 200 ng/mL). Cumulative data of average % cell viability (±SD) with N=3., DMEM; Dulbecco’s Modified Eagle’s Medium.

## Description

According to recent statistics, breast cancer has been one of the most commonly diagnosed cancers among women in the United States (Siegel, Miller, & Jemal, 2019) . Numerous risk factors such as alcohol use, obesity, family history, hormonal therapy, diet, and age, etc. have been associated with breast cancer development (Akram, Iqbal, Daniyal, & Khan, 2017; Wood, Cuke, & Bedrosian, 2019) . Even though several treatment plans have been developed, complete cure for metastatic breast cancer is lacking due to poor prognosis (Peart, 2017) . Although various types of breast cancer cells have been used in research laboratories, MCF-7 cells are perhaps one of the most well studied cell lines (Jiang et al., 2016) . The name of MCF-7 is derived from the Michigan Cancer Foundation, which were isolated from a 69-year-old woman with metastasis (Comsa, Cimpean, & Raica, 2015). MCF-7 cells are estrogen receptor (ER)-positive and progesterone receptor (PR)-positive cells with metastatic potential. The metastasis is developed by the secretion of vascular endothelial growth factor (VEGF-A) by MCF-7 cells and the VEGF induces migration and invasion of the cancer cells (Comsa et al., 2015). Using this cell line, researchers have found effective pharmacological targets to treat breast cancer. While there are some treatment options for breast cancer patients, strategies to prevent the disease altogether is the most effective way to deal with it, and one of the ways to prevent cancer is by regulating diet (De Cicco et al., 2019) .

Throughout the world, people have looked for and consumed natural diets due to health benefits they provide. A popular natural food is bitter melon. Bitter melon grows in tropical and subtropical areas such as Asia, mostly in India and Southeast Asia, Africa, the Caribbean, and California, Florida, and Texas in the United States (Pahlavani et al., 2019; Perez, Jayaprakasha, Crosby, & Patil, 2019; Raina, Kumar, & Agarwal, 2016) . There are multiple ways to consume bitter melon: bitter melon juice or tea, salad, and stir-fry, and it has been used traditionally as folk medicine as well (Raina et al., 2016) . For this reason, many researchers have paid attention to its health benefits, and they have investigated the effect of bitter melon on various cell functions using *in vitro* and *in vivo* study models. Researchers have found that BME inhibits the expression of cell cycle regulatory proteins such as cyclin B1 and D1, blocks G2-M transition of the cell cycle, and thus inducing apoptosis in cancer cells (Cao et al., 2015; Ray, Raychoudhuri, Steele, & Nerurkar, 2010) . While anti-proliferative, proapoptotic, and autophagic effects of bitter melon were found in previous studies, these studies did not use the entire fruit of bitter melon when studying the anti-cancer effects of bitter melon on MCF-7 cells. Instead, deseeded bitter melon extract (BME) or any isolated compounds from bitter melon was used (Bai et al., 2016; Cao et al., 2015; Grossmann et al., 2009; Muhammad, Steele, Isbell, Philips, & Ray, 2017; Ray et al., 2010; Weng et al., 2013). In reality, people do not just eat isolated compounds of bitter melon or deseeded fruit; rather, they usually consume the entire fruit of bitter melon. Therefore, it is important to determine the effects of whole bitter melon extract on MCF-7 cells. We investigated the role of bitter melon extract from the entire fruit including seeds in this study. Furthermore, whether the effects of bitter melon on cellular functions is dose dependent has not been well established.

We hypothesized that the juice extracted from whole bitter melon fruit (with seeds in the bitter melon) will exert cytotoxic effects on MCF-7 cells, similar to the effects observed from deseeded bitter melon or isolated compounds of bitter melon. To test this hypothesis, we co-cultured MCF-7 cells with increasing doses of BME followed by viability assays and microscopic imaging of cells. MCF-7 cells viability was decreased by BME in a dose dependent manner ( Figure 1A ). Microscopic imaging of cell culture revealed dose-dependent decline in the number of cells remaining in the culture dish ( Figure 1B ). The conditioned media from these cultures were separated and glucose concentration was measured in them. The cultures treated with the highest dose of BME that resulted in the least viability of MCF-7 cells ( Figure 1A ) had the highest concentration of remaining glucose in the conditioned media ( Figure 1C ). Reciprocally, the cultures treated with the lowest dose of BME that resulted in relatively the highest cell viability ( Figure 1A ) had the lowest concentration of remaining glucose in the medium ( Figure 1C ). These observations suggest that BME exerts cytotoxic effects on cells in a dose dependent manner. Furthermore, stronger cytotoxic effects on MCF-7 cells may lead to less number of remaining cells, which in turn likely results into less glucose clearance from the culture medium. Our findings in cancer cells is interesting compared to the effects of BME in glucose clearance by the healthy tissues (skeletal muscles and adipose tissues) reported by others (Ma, Yu, Xiao, & Wang, 2017; Nkambo, Anyama, & Onegi, 2013) (Shih, Lin, Lin, & Wu, 2009) (Roffey, Atwal, Johns, & Kubow, 2007) . Both the *in vitro* and *in vivo* studies have reported that BME enhances glucose clearance from the extracellular fluid (culture medium or blood) by the healthy skeletal muscle cells or adipose cells. Thus, BME was reported to lower the blood glucose levels by increasing GLUT-4 transporter mediated downstream signaling pathways resulting in enhanced glucose clearance from blood. In the major insulin sensitive healthy tissues, insulin stimulates glucose uptake by enhancing mobilization of endocytosed GLUT-4 transporters to the cell membrane, which then facilitates glucose transport into the cells, a vital biochemical process for the metabolism & viability of mammalian cells. Thus, in our study, we wanted to test if enhancing glucose uptake by the MCF-7 cells would increase their viability under basal or stressed conditions. To the best of our knowledge, effects of altering glucose uptake by the MCF-7 cells on these cells’ viability have not been investigated. We used insulin to enhance glucose uptake by these cells and test if insulin supplementation will rescue BME’s cytotoxic effects in MCF-7 cell cultures. We hypothesized that insulin supplementation will rescue BME induced toxicity on MCF-7 cells. To test this hypothesis, we co-cultured MCF-7 cells with 2% BME & three different doses of insulin (50, 100, and 200 ng/mL). From the BME dose response experiments, 5% and 10% BME exerted such a strong cytotoxic effects that we observed almost zero viability of the MCF-7 cells in culture ( Figure 1B ). Thus, we decided to use a middle dose of BME (2%) for our rescue experiments. The higher dose of insulin alone (200 ng/mL), with no BME added, neither inhibited nor activated the cells cultured in high glucose medium ( Figure 1D ). However, we found that the insulin supplementation rescued the BME-induced cytotoxicity ( Figure 1D ) most likely by increasing glucose uptake by the cells. Among the three doses of insulin (50, 100, and 200 ng/mL), 50 ng/mL insulin rescued the cell toxicity close to normal viability. To our surprise, compared to 50 ng/mL insulin, higher doses of insulin (100, and 200 ng/mL) did not show a cumulative increase in the rescue effect ( Figure 1D ). Interestingly, at doses higher than 50 ng/mL, insulin appeared to show decreasing effects ( Figure 1D ). We do not know why higher doses of insulin become less effective in rescuing the cells from the BME’s cytotoxic effects.

One possibility could be that maximal glucose uptake is achieved by the 50 ng/mL insulin. Furthermore, higher doses of insulin may exert suppressive effects on cells-a phenomenon named as insulin toxicity (Kolb, Kempf, Rohling, & Martin, 2020) . It appears that when cells are exposed to higher doses of insulin, downregulation of insulin signaling occurs, which is not primarily due to less insulin receptor expression on the cell surface but due to impaired insulin signal transduction as a result of receptor dysfunction (Bertacca et al., 2005; Catalano et al., 2014) . Bertacca and colleagues have reported that higher dose of insulin downregulates GLUT4 receptor expression on the cell surface (Bertacca et al., 2005) . Downregulation of GLUT4 receptor is likely to add stress to the cells as they will not be efficient to take up glucose from the medium. Thus, the cells co-cultured with high insulin dose in a low glucose medium (100 mg/dL glucose compared to high glucose medium with 450 mg/dL glucose) are likely to experience double stress due to downregulation of GLUT4 receptors and low glucose environment. To further evaluate this possibility, we repeated the rescue experiments in low glucose DMEM medium in which glucose concentration is approximately five-fold lower. In the low glucose medium, concentration gradient of glucose between the culture medium and cell cytoplasm decreases. Thus, low glucose medium is likely to result into less glucose influx into the cells, which in turn can induce stress and thereby decrease viability in cells. We found that in contrast to the cells cultured in high glucose DMEM medium, cells cultured in low glucose DMEM medium lost rescue sensitivity to 200 ng/mL insulin. Moreover, higher dose of insulin alone (200 ng/mL), with no BME added, neither inhibited nor activated the cells cultured in low glucose medium ( Figure 1E ), similar to what we found in high glucose medium culture ( Figure 1D ). We do not know why 200 ng/mL insulin alone is neither beneficial nor harmful to cells.

One of the limitations of this study is that we do not know if the BME (obtained from whole fruit with seeds in it) inhibits glucose uptake by the MCF-7 cells or glucose clearance from the medium is suppressed as a secondary effect of BME’s cytotoxicity on MCF-7 cells. Cancer cells express different subtypes of GLUT transporters (GLUT1, GLUT2, GLUT3, GLUT4 etc.) (Ancey, Contat, & Meylan, 2018) . Previous studies have emphasized that ubiquitous GLUT-1 transporter plays major roles in glucose uptake in cancer cells (Shin & Koo, 2021) . However, whether relative contributions of GLUT-1 and GLUT-4 transporters are different in MCF-7 cells under healthy versus stressful situations is not known. Our findings suggest that insulin dependent glucose uptake is critical to overcome stressors such as BME induced cytotoxicity. Our findings also suggest that the rescue effect of insulin is dependent on the glucose concentration in the media, particularly at higher doses of insulin ( Figure 1D and Figure 1E). Studies have found overexpression of IGF-1 receptor in metastatic breast cancer cells compared to the normal cells (Bartucci, Morelli, Mauro, Ando, & Surmacz, 2001; Cullen et al., 1990; Surmacz, Guvakova, Nolan, Nicosia, & Sciacca, 1998) . Furthermore, insulin and IGF-1 ligands can potentially cross stimulate their receptors (Griffeth, Bianda, & Nef, 2014) . Therefore, enhanced glucose uptake could also be secondary to the signaling pathways associated with IGF-1 receptor stimulation and not necessarily via the well-established insulin-GLUT-4 pathway. Several of these possibilities could be investigated in the future.

## Methods

**Bitter Melon Extract (BME) Preparation**

Fresh bitter melons were purchased from an Asian grocery store. The whole bitter melon (with seeds in them) were washed and cut into small pieces to fit in the juice extractor (Black & Decker; Product # JE2400BD). The pieces of the entire fruits of bitter melons, seeds included, were used. The juice extract was centrifuged nine times for 15 minutes each at 5000 rpm, and then filter sterilized. BME aliquots were stored at - 80°C.

**Cell culture & BME dose response**

Equal number of MCF-7 cells (1.0 x 10 ^5^ viable cells/cm; Thermo Fisher Scientific, catalog # HTB-22) were plated in the 75 cm ^2^ tissue culture treated culture flasks (Thermo Fisher Scientific, catalog # 156499) containing 30 mL medium (Millipore Sigma, DMEM-high glucose, catalog # D6429). The medium was supplemented with 10% Fetal Bovine Serum (Millipore Sigma, catalog # F2442). Any other appropriate treatments such as BME, insulin etc were added to the medium just before adding the cell inoculum to the complete growth medium. Cells were incubated in an incubator (37˚C, 5% CO _2_ , 0% O _2_ ) for 48 hours. After 48 hours, cultures were washed with 1X phosphate buffered saline (Millipore Sigma, catalog # TMS-012) followed by capturing of pictures using a microscope at the 40x magnification (Nikon, product # 25726). The glucose concentration remaining in the culture medium was also measured. Cell viability was also measured using Trypan Blue Exclusion assay.

**Glucose measurement**

After culturing cells for 48 hours, culture media were removed and centrifuged at 2000 rpm for 3 minutes, and then the media were transferred to sterile centrifuge tubes to measure the glucose levels from each tube using a glucometer (BioReactor Sciences, Model # GM100).

**Trypan blue exclusion assay**

After MCF-7 cells were cultured with the treatments for the required amount of time, the supernatant was collected from each culture flask. After supernatant was collected from each culture flask, the cells were washed with 1X PBS three times, and they were trypsinized from the bottom of the culture flasks with Trypsin-EDTA (0.25%), phenol red (catalog # 25200056). Trypsinized cells were combined with cells from the supernatant. The cells were resuspended in 1 mL DMEM (Millipore Sigma, DMEM-high glucose, catalog # D6429) and mixed softly with pipette. 20 μL cells from the cell suspension were added to a cryovial. Equal parts of 0.4% trypan blue solution (VWR, CAS # 72-57-1) were added to the cryovial containing the cells to obtain a 1 to 1 dilution, and 9 μL of the solution was pipetted from the cryovial and loaded into the hemocytometer chamber (Fisher Scientific, catalog # 02-671-55A). Then, cells were counted using a microscope at total magnification of 100X. Both total number of cells and blue-stained cells were counted from the four chamber of the hemocytometer, and number of blue-stained cells were subtracted from the total number of cells to give the number of viable cells. After that, the average was taken from the number of viable cells, and then it was multiplied by 10,000 which represented the number of cells per mL and by a dilution factor, thus giving the number of viable cells per mL.

**MTT; 3-(4,5-Dimethylthiazol-2-yl)-2,5-Diphenyltetrazolium Bromide (MTT) assay**

The cells were seeded at a concentration of 0.8 × 10 ^5^ cells/mL into a 96-well plate. The cells were incubated (37˚ C, 5% CO _2_ , 0% O _2_ ) with the medium alone or with the appropriate supplements (different doses of BME, different doses of insulin or both insulin and 2% BME) in the medium. After culturing cells for 48 hours, medium was aspirated from each well and 100 μL fresh medium containing DMEM and 10% FBS was added to the wells. Then 10 μL of MTT reagent (Millipore Sigma, catalog # 11465007001) was added to the wells. The contents of wells in the 96-well plate were mixed using a shaker followed by incubation of the plate (37˚ C, 5% CO _2_ , 0% O _2_ ) for 4 hours. After 4 hours, 100 μL of detergent (0.01N HCl with 10% sodium dodecyl sulfate) was added to each well and the plate was incubated in dark at room temperature for four hours. After the incubation, the plate was read using spectrophotometer plate reader (Molecular Devices; Filter Max F3 Multi-Mode Microplate Reader) and a reading software (SoftMax Pro 6.2.1), and absorbance for each well was recorded at 570nm. The cells viability was estimated by measuring absorbance at 570 nm. The cell viability percentage was calculated based on the absorbance ratio between culture wells with different treatments and the untreated control well multiplied by 100 (percentage of control, %).
